# Treatment of Neuromyelitis Optica Spectrum Disorders

**DOI:** 10.3390/ijms22168638

**Published:** 2021-08-11

**Authors:** Koon-Ho Chan, Chi-Yan Lee

**Affiliations:** 1 Department of Medicine, LKS Faculty of Medicine, The University of Hong Kong, Hong Kong, China; cylee@gmail.com; 2 Neuroimmunology and Neuroinflammation Research Laboratory, LKS Faculty of Medicine, The University of Hong Kong, Hong Kong, China; 3 Research Center of Heart, Brain, Hormone and Healthy Aging, LKS Faculty of Medicine, The University of Hong Kong, Hong Kong, China

**Keywords:** neuromyelitis optica spectrum disorders, aquaporin-4 autoimmunity, AQP4-IgG, B lymphocytes, T lymphocytes, immunosuppressive therapies

## Abstract

Neuromyelitis optica spectrum disorder (NMOSD) is an autoimmune central nervous system (CNS) inflammatory disorder that can lead to serious disability and mortality. Females are predominantly affected, including those within the reproductive age. Most patients develop relapsing attacks of optic neuritis; longitudinally extensive transverse myelitis; and encephalitis, especially brainstem encephalitis. The majority of NMOSD patients are seropositive for IgG autoantibodies against the water channel protein aquaporin-4 (AQP4-IgG), reflecting underlying aquaporin-4 autoimmunity. Histological findings of the affected CNS tissues of patients from in-vitro and in-vivo studies support that AQP4-IgG is directly pathogenic in NMOSD. It is believed that the binding of AQP4-IgG to CNS aquaporin-4 (abundantly expressed at the endfoot processes of astrocytes) triggers astrocytopathy and neuroinflammation, resulting in acute attacks. These attacks of neuroinflammation can lead to pathologies, including aquaporin-4 loss, astrocytic activation, injury and loss, glutamate excitotoxicity, microglial activation, neuroinflammation, demyelination, and neuronal injury, via both complement-dependent and complement-independent pathophysiological mechanisms. With the increased understanding of these mechanisms underlying this serious autoimmune astrocytopathy, effective treatments for both active attacks and long-term immunosuppression to prevent relapses in NMOSD are increasingly available based on the evidence from retrospective observational data and prospective clinical trials. Knowledge on the indications and potential side effects of these medications are essential for a clear evaluation of the potential benefits and risks to NMOSD patients in a personalized manner. Special issues such as pregnancy and the coexistence of other autoimmune diseases require additional concern and meticulous care. Future directions include the identification of clinically useful biomarkers for the prediction of relapse and monitoring of the therapeutic response, as well as the development of effective medications with minimal side effects, especially opportunistic infections complicated by long-term immunosuppression.

## 1. Introduction

Neuromyelitis optica spectrum disorder (NMOSD) is a central nervous system (CNS) autoimmune inflammatory demyelinating disorder causing blindness, paralysis, cognitive impairment, and even mortality. Most patients develop relapsing attacks of CNS inflammation, typically optic neuritis; longitudinally extensive transverse myelitis (LETM); and, less frequently, encephalitis affecting the diencephalon, area postrema, and other brainstem regions [1,2,3,4,5,6,7]. The majority of NMOSD patients have a relapsing rather than monophasic disease course [1,2,3]. Classical NMOSD patients have severe neurological disability after attacks of LETM, severe ON, no secondary progression, and worse clinical outcomes than multiple sclerosis patients, and cerebrospinal fluid (CSF) oligoclonal bands are infrequently detected in about 30% of patients [1,2,3,4]. Importantly, about 75–80% of NMOSD patients are seropositive for immunoglobulin G (IgG) autoantibodies specific for aquaporin-4 (AQP4-IgG) [1,2,3,8,9]. Aquaporin-4 (AQP4) is a transmembrane water channel highly expressed in the endfeet of CNS astrocytes, which is essential for normal functioning of the blood−brain barrier (BBB) and for maintenance of CNS water homeostasis [2]. AQP4-IgG is detected in the serum of a AQP4-IgG positive NMOSD patient, only rarely in CSF [2,3]. It is now clear that NMOSD seropositive for AQP4-IgG is not multiple sclerosis, but an autoimmune disorder affecting predominantly CNS astrocytes (astrocytopathy), and the detection of AQP4-IgG in the serum of patients greatly facilitates the diagnosis of NMOSD. NMOSD patients seropositive for AQP4-IgG have underlying AQP4 autoimmunity [1,2,3,8,9]. A small proportion of patients with typical neurological features of NMOSD are seronegative for AQP4-IgG, and their pathogenesis is uncertain. Some of these AQP4-IgG-seronegative NMOSD patients, 10–40%, are seropositive for IgG autoantibodies against myelin oligodendrocyte glycoprotein (MOG-IgG) [10]. The pathogenesis of MOG-IgG-seropositive patients awaits clarification. Converging lines of evidence support that CNS inflammatory demyelinating disorders associated with MOG-IgG have different pathophysiologies and pathologies from AQP4-IgG-seropositive NMOSD, and are considered as a separate entity termed myelin oligodendrocyte glycoprotein antibody associated disease (MOGAD), which phenotypically can mimic NMOSD [10]. In this review, seronegative NMOSD patients refer to those with neurological features fulfilling the 2015 diagnostic criteria for seronegative NMOSD, who were seronegative for both AQP4-IgG and MOG-IgG by cell-based assays [5]. In this review, we focus on the treatments of NMOSD only, not MOGAD.

## 2. Pathophysiologies

### 2.1. Aquaporin-4 Autoimmunity

Early histological studies showed that spinal cord tissues of NMOSD patients exhibit necrosis in both gray and white matter, infiltrating leucocytes (including macrophages, neutrophils, eosinophils, and lymphocytes) and activating microglia; demyelination; axonal loss; thickened hyalinized vessel walls with deposits of IgM, IgG, and complement activation products in a vasculocentric rim and rosette pattern. This strongly suggests the pathogenic role of autoantibodies and complements activation in the neuroinflammation and necrosis of CNS tissues in NMOSD [11]. After the discovery of AQP4-IgG, histological studies further revealed that the CNS lesions of patients demonstrated significant AQP4 loss with or without astrocyte loss [12]. These hyalinized vessel walls with deposits of immunoglobulins and complement activation products in the characteristic vasculocentric rim and rosette pattern, and AQP4 loss, are not observed in the lesions of multiple sclerosis patients [11,12]. These histopathological observations of CNS lesions of patients, together with in-vitro and in-vivo studies of the pathogenicity of AQP4-IgG, strongly support that AQP4-IgG is directly pathogenic in NMOSD [2,3,12,13]. It is believed that circulating AQP4-IgG triggers attacks of neuroinflammation when it gains access to the CNS, (1) when the BBB is breached (for example by proinflammatory cytokines during infections) or (2) at the area postrema of the brainstem lacking an intact BBB and trigger area postrema syndrome, characterized by persistent hiccups and vomiting (NMOSD patients can develop optic neuritis or myelitis preceded by area postrema syndrome) [2,3,14]. The binding of AQP4-IgG to astrocytic AQP4 can trigger acute attacks of neuroinflammation and induce CNS pathologies, including astrocytopathy, astrocyte loss via complement activation (complement-dependent cytotoxicity) and antibody-dependent cellular cytotoxicity, BBB disruption, neuroinflammation, demyelination, and neuronal injury [2,12]. A wide spectrum of pathologies have been described in NMOSD patients, which have been classified into six different lesion types, suggesting that acute attacks of neuroinflammation involve complex and multiple mechanisms of tissue injury, including both complement-dependent and complement-independent mechanisms [15].

### 2.2. Complement-Dependent Pathophysiologies

AQP4-IgG belongs to the IgG1 subclass that is complement-activating [13]. Complement activation is clearly important in NMOSD pathophysiologies, leading to the formation of the membrane attack complex, which mediates astrocyte cytotoxicity via pore formation on the cell membrane (complement dependent cytotoxicity), and complement activation products such as C3a and C5a, which mediate and amplify neuroinflammation via their chemotactic effects with the infiltration of neutrophils, eosinophils, and macrophages into the CNS tissues [2,12,16].

Aquaporin-4 is a bidirectional transmembrane water channel predominantly expressed by astrocytes at their endfeet processes, but also by ependymal cells in the CNS. The most common isoforms of aquaporin-4 in the CNS are the M1 and M23 isoforms, and their relative abundance may contribute to differences in lesion pathology and disease severity [2,17]. Aquaporin-4 exists as homotetramers or heterotetramers in the cell membrane. With the increased abundance of the M23 isoform, a large orthogonal array of particles (OAP) will be formed by tetramer aggregation and assembly on the cell membrane, while a few OAP will be present if the M1 isoform is predominant, as the M1 isoform cannot assemble to form OAP. It is believed that complement activation after AQP4-IgG binding to aquaporin-4 requires aquaporin-4 supermolecular clustering in the OAP [18,19], whereas in the absence of OAP, AQP4-IgG binding to aquaporin-4 leads to internalization of the antigen−autoantibody complex, followed by endolysosomal degradation or direct blockade of the water channel leading to impaired water transport and water dyshomeostasis with oedema [13]. Although aquaporin-4 is also expressed in the cells of other organs, including the retina, gastric and bronchial mucosa, renal tubules, and skeletal muscle, pathology affecting these organs are rare. This may be explained by different expression levels of the M1 and M23 isoforms [2,17], as well as complement regulatory proteins in different regions and organs [2,20].

### 2.3. Complement-Independent Pathophysiologies

Normal CNS lacks the initiator of complement cascade C1q; hence, the initial access of AQP4-IgG to CNS and the binding to astrocytic AQP4 do not activate the complements. Complement-independent mechanisms are important for NMOSD pathophysiologies, especially for the early development of lesions [21]. AQP4-IgG-induced astrocytic activation leads to the upregulation and secretion of proinflammatory cytokines, chemokines, and other mediators of inflammation and oxidative stress [22]. These inflammatory mediators can lead to microglial activation and proliferation, macrophage activation, and neutrophil/eosinophil infiltration observed in the affected CNS tissues of patients. Prominent microglial activation is observed in the area postrema, other brainstem regions, and spinal cord lesions of patients with NMOSD [12,14]. Astrocyte loss in NMOSD is likely also mediated by antibody-dependent cell-mediated cytotoxicity (ADCC), predominantly via activated microglia and macrophages that express Fc receptors for IgG binding [23]. Fc receptors binding to the Fc portion of AQP4-IgG bound to astrocytes trigger a release of cytotoxic compounds and astrocytic phagocytosis. In vitro and in vivo studies support that ADCC contributes to AQP4-IgG-induced astrocyte loss in NMOSD lesions [21,24]. The binding of AQP4-IgG to astrocytes enhances BBB permeability, leading to natural killer cell degranulation and astrocyte cytotoxicity in vitro [25]. Mice treated with AQP4-IgG incapable of ADCC activation and human complement have attenuated NMOSD-like pathologies [26]. Chronic infusion of AQP4-IgG intrathecally to rats resulted in astrocyte damage associated with myelin changes, loss of axons and oligodendrocytes, and motor impairment, without complement activation and immune cell infiltration [27]. Direct AQP4-IgG-mediated astrocytopathy, without the involvement of immune cells and complements, may lead to astrocytic loss. These complement-independent pathopathologies, including astrocytopathy and neuroinflammation, may lead to a further breakdown of BBB [28] so as to facilitate the entry of AQP4-IgG and complements to the CNS, which then trigger severe neuroinflammation and CNS damage such as necrosis via complement activation [29].

Other mechanisms of AQP4-IgG-induced astrocyte loss may include glutamate excitotoxicity, as astrocytes also express glutamate receptors [2,3,10,30]. AQP4 is physiologically coupled with the glutamate transporter EAAT2 in the astrocytic membrane. Without complements, cultured astrocytes exposed to NMOSD patient serum have AQP4 endocytosis with concomitant loss of EAAT2 and a reduction of glutamate reuptake [31]. In cultured astrocytes and oligodendrocytes exposed to IgG from AQP4-IgG-seropositive patients, the activity of astrocytic glutamine synthase decreased in parallel with the progressive accumulation of glutamate in the culture medium, loss of oligodendritic cell processes, and oligodendrocyte cytotoxicity [32]. Oligodendrocytic injury and loss can contribute to the demyelination observed in NMOSD lesions, in addition to activated microglia/macrophage-mediated demyelination [33].

In AQP4-IgG-seropositive NMOSD patients, AQP4-IgG is detected in the peripheral blood, but not in CSF, in the majority of patients [2,3]. The mechanism underlying entry of peripheral AQP4-IgG into the CNS in NMOSD is unclear. Viral infections have been observed to precede attack in 25% of NMOSD patients [4]. Systemic infections can trigger the breakdown of BBB via increased levels of circulating proinflammatory cytokines [29], followed by the entry of AQP4-IgG and the development of an acute attack. The proinflammatory cytokines include IL-1β, IL-6, and TNFα; of these, IL-6 is highly relevant in NMOSD, as IL-6 has been demonstrated to promote the survival of peripheral blood plasmablasts (antibody-secreting cells) and their secretion of AQP4-IgG in AQP4-IgG-seropositive NMOSD patients [34]. In addition, autoantibodies targeting the brain microvascular endothelial cells, including glucose-regulated protein 78 (GRP78) autoantibodies, have been identified in the sera of AQP4-IgG-seropositive NMOSD patients, which disrupt the BBB and increase BBB permeability via the upregulation of the vascular endothelial growth factor (VEGF) and downregulation of the tight junction proteins such as claudin-5 [35,36,37]. These autoantibodies may play an important role in the development of acute attacks in NMOSD.

### 2.4. Role of B Cells in AQP4-IgG Positive NMOSD

AQP4-IgG positive NMOSD patients have underlying autoimmunity against AQP4. Although the etiology and mechanisms triggering AQP4 autoimmunity and the production of AQP4-IgG autoantibodies in NMOSD are uncertain, B cells definitely have important roles in the pathophysiologies, as the production of pathogenic high affinity AQP4-IgG requires memory B cells autoreactive to AQP4 to differentiate to plasmablasts and mature plasma cells (antibody secreting cells (ASC)), which produce AQP4-IgG [38]. Plasmablasts are increased in the peripheral blood of NMOSD patients, which secrete AQP4-IgG driven by interleukin-6 (IL-6) [34]. Memory B cells (Bmems) specific for AQP4 are likely crucial in AQP4 autoimmunity. Memory B cells are considered as a source of proinflammatory cytokines responsible for pathogenic effects in autoimmune diseases [38]. As rituximab is effective in NMOSD patients without a significant fall in serum AQP4-IgG titres, B cells probably contribute to NMOSD pathophysiologies via other mechanisms, including antigen presentation to activate T cells and the secretion of proinflammatory cytokines [38]. Wilson et al. showed that peripheral blood B cells isolated from AQP4-IgG positive NMOSD patients can be cultured to differentiate to ASC (CD19+CD27++CD38++), which secrete AQP4-IgG in the absence of the autoantigen AQP4 with a combination of cytokines including IL-1β, IL-2, IL-6, IL-21, and TNFα, and toll-like receptor agonist [39]. This confirms the presence of AQP4 autoreactive B cells that differentiate to ASC capable of producing AQP4-IgG in the absence of AQP4. Although both naïve B cells (pre-germinal center), and unswitched and switched memory B cells (post-germinal center) cultured ex vivo could produce AQP4-IgG, switched memory B cells have a much higher capacity of AQP4-IgG production [39].

There are two tolerance checkpoints for B cell development—a central tolerance checkpoint in the bone marrow for development from early immature B cells to immature B cells, and a peripheral tolerance checkpoint from development of new emigrant B cells to mature naïve B cells [38]. As an autoantibody-mediated autoimmune disease, the exact immune checkpoints where loss of immune tolerance to AQP4 in AQP4-IgG positive NMOSD occur are uncertain. Cotzomi et al. identified defects in both the central and peripheral B cell tolerance checkpoints in NMOSD, as higher frequencies of polyreactive and autoreactive new emigrant/transitional and mature naïve B cells were found in the peripheral blood of NMOSD patients compared with that of the healthy controls. The investigators further studied the revertants of AQP4-IgG (AQP4-IgG with acquired somatic mutations reverted back to unmutated germline precursors) and revealed that somatic hypermutation is required for the generation of AQP4-IgG, as all reverted unmutated germline precursors did not bind to AQP4. However, one-third and two-thirds of the revertants were polyreactive and autoreactive, respectively, suggesting that pathogenic AQP4-IgG can originate from the pool of autoreactive naïve B cells that develop as a consequence of impaired early B cell tolerance checkpoints in NMOSD patients [40].

In addition, the functions of regulatory B cells (Bregs) are impaired in NMOSD patients, especially with active disease [41,42]. Bregs are indispensable for the maintenance of self-tolerance and immune homeostasis [43]. Altered number and functions of memory B cells and Bregs in NMOSD have been reported. Quan et. al. showed that CD19^+^CD24^hi^CD38^hi^ Breg levels and B cell expression of IL-10 were significantly lower in NMOSD patients than in healthy controls, and B cell IL-10 production and CD19^+^CD24^hi^CD38^hi^ regulatory B cell levels in AQP4-IgG-seropositive patients were even lower than that in AQP4-IgG-negative patients [41]. The same group further reported that CD19^+^CD24^+^CD38^+^ Breg had an impaired function to secrete of IL-10, whereas memory B cells (CD19^+^CD27^+^) were enhanced in their function to secrete the proinflammatory cytokine (IFNγ) in NMOSD patients [42]. The immunoregulatory properties of B cells are likely impaired in AQP4-IgG-seropositive NMOSD patients.

### 2.5. Role of T Cells and B Cell-T Cell Interaction in AQP4-IgG Positive NMOSD

AQP4-IgG belongs to the IgG1 subclass that requires T cell help for development and affinity maturation [2,3]. T cells also play important roles in AQP4-IgG positive NMOSD. AQP4 autoreactive T cells are identified in NMOSD patients, which are polarized to the Th17 phenotype, with the immunodominant T cell epitope having a high degree of homology to Clostridium perfringens ABC transporter [44]. Studies based on the immunization of wild type and AQP4 deficient mice with immunodominant AQP4 peptides suggest that impaired central (thymic) immune tolerance (deletional tolerance) contributes to pathogenic AQP4-specific T cell responses [45]. AQP4 autoreactive B cells likely internalize AQP4 bound to their B cell receptor (BCR), process AQP4 (autoantigen), and then present it via major histocompatibility complex class II (MHC II) to T cells in the germinal centers of secondary lymphoid organs. These activated T cells develop into AQP4 autoreactive CD4+ T follicular help cells (Tfh), which are critical for differentiation, somatic hypermutation, class switching, and affinity maturation of B cells into AQP4 autoreactive class-switched memory B cells, plasmablasts, and plasma cells (ASC), which secrete pathogenic AQP4-IgG [46]. IL-21 produced by Tfh is the key cytokine for B cell development and maturation [46]. Most recently, several subtypes of circulating Tfh cells, including Tfh1, Tfh2, and Tfh17, together with T follicular regulatory (Tfr), were identified in the peripheral blood of NMOSD patients. Tfh2 and Tfh17 cells strongly support B cell differentiation, whereas Tfh1 do not, and Tfr inhibit B cell differentiation. Importantly, untreated NMOSD patients had Tfh polarization towards excessive B-helper Tfh subsets (increase of Tfh17 and (Tfh2 + Tfh17)/Tfh1 ratio and decrease of Tfr and Tfh1), which was restored by rituximab [47].

## 3. Treatments for Acute Attacks of NMOSD

NMOSD mostly has a relapsing course if left untreated [4]. The commonly accepted definition of an acute NMOSD attack follows that of multiple sclerosis (MS): new or worsening neurological symptoms lasting for at least 24 h without an alternative explanation (e.g., fever and infection) and occurring more than 30 days after the previous attack [48]. Compared with MS, relapses in NMOSD tend to be more severe and only partially reversible, and may lead to visual loss, paralysis, bladder dysfunction, neuropathic pain, altered conscious level, and even respiratory failure and mortality. In addition, disability accumulation in NMOSD is attributed to incomplete recovery from each relapsing attack, rather than secondary progression or degeneration [49]. It has been reported that a mean of three spinal attacks could result in paraplegia, whereas a mean of 1.5 optic attacks could result in blindness [50,51].

The socioeconomic burden of relapses and long-term disease-related care is substantial. In a cohort of 1363 patients with NMOSD in the US, 47.7% experienced one or more relapses during a median follow up period of two years [52]. The average healthcare cost among patients with NMOSD was $60,599 per year compared with $8912 per year for patients without NMOSD [52]. Apart from preventive therapies to reduce relapses, prompt and efficacious treatment of acute attacks is crucial to hasten recovery, reduce neurological damage, improve functional outcomes, and lower healthcare expenditure.

Table 1 lists the treatments available for acute attacks of NMOSD. High dose intravenous steroids are traditionally used as first-line treatment of acute attacks of NMOSD, typically intravenous methylprednisolone (IVMP) at 1 g per day for 3–5 consecutive days. The proposed mechanisms of IVMP include the (i) inhibition of pro-inflammatory cytokine production, (ii) downregulation of expression of cell adhesion molecules and receptors, (iii) augmentation of anti-inflammatory cytokine secretion, (iv) reduction and modulation of T-cell activity by inducing T cell apoptosis and causing expansion of myeloid-derived suppressor cells, (v) restoration of the integrity of the blood–brain barrier by downregulating the matrix metalloproteinases, and (vi) repression of nitric oxide production by myeloid cells [53]. Side effects include hypertension, hyperglycemia, euphoria, arrhythmia, peptic ulcer, osteoporosis, and avascular necrosis of the femoral head.

Recent studies have shown that IVMP could improve visual acuity and preserve retinal nerve fiber layer thickness in optic neuritis associated with NMOSD, and a delay in IVMP treatment is associated with adverse visual outcomes [54,55]. However, in a study including 693 NMOSD attacks in 181 patients, only 17.0% achieved complete remission and 16.2% had no improvement after receiving IVMP as the first treatment [56].

Plasma exchange (PLEX) has been shown to improve neurological functions in patients with severe attacks of central nervous system inflammatory demyelinating disorders who failed standard treatment with high dose intravenous steroid in a randomized sham-controlled trial [57]. Possible mechanisms of PLEX include removal of the autoantibodies and proinflammatory factors, and regulation of the lymphocytic function and proliferation [58]. A typical regimen is five to seven sessions of PLEX on every other day, with 2 L or up to 1.5 total plasma volume of replacement fluid (e.g., albumin or plasma) in each session. Potential side effects of PLEX include catheter-related infections and complications, hypotension, coagulopathies, and electrolyte disturbances.

Several studies have confirmed the efficacy of PLEX as a rescue therapy to corticosteroid-resistant relapses, specifically in patients with NMOSD [59,60,61,62,63]. Other studies have shown the efficacy of PLEX as an add-on treatment to IVMP to reduce disability in severe spinal attacks [64] and to improve visual acuity in optic neuritis [65,66] in NMOSD, compared with the use of IVMP as the only treatment. There is also plausible evidence that an earlier initiation of PLEX is associated with a better response and functional outcome [48,51,56,67,68]. In summary, PLEX should be started early in patients who show suboptimal improvement with IVMP, and concomitant use of IVMP and PLEX may be considered as an initial treatment in severe attacks of NMOSD.

Immunoadsorption (IA) is an alternative apheresis therapy to PLEX when PLEX is contraindicated or unavailable [69]. During IA, the plasma fraction is separated and then passed through an IA device where tryptophan or protein A is used as an absorber. This process provides a rapid removal of immunoglobulins and complements, while the albumin and clotting factors are mostly preserved [70]. PLEX and IA were shown to be similarly effective in NMOSD attacks [48]. There has been no study to directly compare the two apheresis therapies to IVMP as an initial therapy, but one study suggested PLEX/IA were superior to high dose steroids as a first treatment in NMOSD attacks with isolated myelitis [56].

Intravenous immunoglobulins (IVIg) therapy is occasionally considered as a treatment option in NMOSD attacks, especially in patients with contraindications to IVMP and apheresis therapies. The potential mechanisms of its immunomodulatory effects include a blockade of cellular receptors, neutralization of cytokines, complements and autoantibodies, and modulation of the immune effector cells [71]. A retrospective review of 10 patients with NMOSD relapses showed effectiveness in IVIg, mainly as a rescue therapy to IVMP [72]. Another study demonstrated adding high dose steroids to IVIg was superior to high dose steroids alone in patients with severe attacks, but did not support the use of IVIg as the only therapy [73]. Further studies are needed to confirm the efficacy of IVIg for the treatment of NMOSD attacks.

Other therapies with different mechanisms under evaluation for the acute treatment of NMOSD relapses include C1-esterase inhibitor, vascular endothelial growth factor, B cells depletion, neonatal Fc receptor inhibitor, and chimeric antigen receptor (CAR) T-cell therapy [74].

## 4. Long-Term Immunosuppressive Treatments to Prevent Relapse in Neuromyelitis Optica Spectrum Disorders

Table 2 lists the treatments available for relapse prevention in NMOSD whereas Table 3 lists the core clinical trials for relapse prevention in NMOSD. 

### 4.1. Conventional Immunosuppressive Medications

#### 4.1.1. Corticosteroids

One study evaluated the efficacy of prednisolone monotherapy and reported the benefit on ARR. Watanabe et al. studied nine NMOSD patients (five AQP4-IgG-seropositive) treated with oral prednisolone (eight periods with >10 mg/day and 18 periods ≤ 10 mg/day for median 19 and 45 months, respectively), the median annualized relapse rate (ARR) was 0.49 and 1.48 during the treated and untreated periods, respectively, and eight patients (89%) had a lower ARR, except one seronegative patient who had more relapses in the <10 mg/day subgroup [75]. Hypertension, diabetes mellitus, osteoporosis, Cushing’s syndrome, and increased risk of infection are common side effects of prolonged corticosteroid therapy. Rare side effects include euphoria and psychosis.

#### 4.1.2. Azathioprine

Azathioprine is a 6-mercaptopurine analogue and suppresses lymphocyte proliferation and activation with anti-inflammatory action. Azathioprine has been used to prevent relapse in NMOSD for decades, since a report on its efficacy in seven NMOSD patients who became relapse free after azathioprine treatment with a significant improvement in neurological function [87]. In a series of 99 patients, among the 70 with a follow-up of ≥12 months, ARR decreased from pre-treatment, 2.2, to post-treatment, 0.52, for those taking azathioprine ≥ 2 mg/kg/day (*n* = 48), whereas ARR decreased from 2.09 to 0.82 for those taking azathioprine < 2 mg/kg/day (*n* = 22). Over a median post-treatment follow-up duration of 22 months, 37% of patients remained relapse free after 2 years of follow-up, with EDSS scores stable or improved despite ongoing relapses in 31% of patients. However, 38 patients (38%) discontinued the drug (due to side effects in 22, no efficacy in 13, and lymphoma in 3) [76]. Similar findings were observed in another retrospective study involving 103 AQP4-IgG-seropositive NMOSD patients, showing that 89% of patients had a significant reduction in median ARR from 1.5 to 0, 61% remained relapse free at a median follow-up of 18 months, and neurological function improved or stabilized in 78% with azathioprine treatment. Similarly, treatment was discontinued in last follow-up in 46% of patients (due to side effects in 62%, deaths in 19%, on-going disease activity in 15%, and pregnancy in 2%). The investigators concluded that azathioprine is a modestly effective treatment for NMOSD, but many patients discontinue over time raising the concern of poor tolerability [77]. Two retrospective studies [88,89] and one small controlled clinical trial [90] have suggested the superiority of rituximab over azathioprine in the prevention of relapse in NMOSD.

Azathioprine is started at 25–50 mg daily and gradually increased to 2–3 mg/kg daily. It may take 4–6 months for effects, and adjunctive oral corticosteroid is usually required during this period. Patients need to be monitored regularly with complete blood count, liver, and renal function tests. The mean corpuscular volume increase has been reported to influence ARR change in NMOSD [76]. Bone marrow suppression resulting in pancytopenia and hepatitis with deranged liver function tests are common side effects. Risk of infection is increased with azathioprine therapy, typically through viral infection such as herpes zoster. Rare severe gastrointestinal symptoms due to allergy to the drug and severe pancreatitis can complicate azathioprine use. Intolerance is not uncommon and long-term therapy bears an increased risk of malignancy, especially for lymphoproliferative disorders.

#### 4.1.3. Mycophenolate Mofetil

Mycophenolate mofetil (MMF), a reversible inhibitor of inosine monophosphate dehydrogenase that is involved in guanosine nucleotide synthesis, reversibly inhibits T and B lymphocyte proliferation. Jacob et al. studied 24 NMOSD patients (seven treatment naïve) treated with MMF at a median dose of 2000 mg per day for a median duration of 27 months, and reported that the median ARR decreased from pre-treatment, 1.3, to post-treatment, 0.09. Nineteen patients (79%) had an improvement in ARR, and disability was stabilized (*n* = 15) or improved (*n* = 7) in 22 (91%). One died of disease complication during follow-up, and adverse effects were reported in six patients (25%), including headache, constipation, easy bruising, anxiety, hair loss, diarrhea, abdominal pain, and leukopenia, which required the discontinuation of MMF treatment [91]. MMF is probably more effective in relapse prevention and also has a better tolerability than azathioprine, but is likely less effective than rituximab, especially in the prevention of severe relapse in NMOSD [88]. It is less expensive than rituximab and is a convenient oral medication. Major concern is teratogenicity with need for contraception in young female patients in reproductive age, and long-term risk of malignancy await clarification. Risk of infection is increased with MMF therapy, including viral infection by herpes viruses and bacterial infection by mycobacteria tuberculosis.

#### 4.1.4. Mitoxantrone, Methotrexate, Cyclosporin, Tacrolimus, and Cyclophosphamide

Mitoxantrone intercalates with the DNA molecule causing single- and double-stranded disruption, and suppresses DNA repair via the inhibition of topoisomerase II. It is a potent immunosuppressant that strongly inhibits the proliferation of T and B cells, as well as macrophages. Three studies reported the therapeutic efficacy of mitoxantrone in preventing relapse and improving or stabilizing the EDSS score in NMOSD—the majority of patients were AQP4-IgG-seropositive [92,93,94]—whereas one retrospective study reported no reduction of relapse rate compared with beta-interferon [95]. The potential serious cardiotoxicity, myelotoxicity, and oncogenicity (treatment related acute leukaemia) of mitoxantrone in the background of increasing the availability of safer and effective treatments such as rituximab and tocilizumab render mitoxantrone use in NMOSD rare and exceptional.

Methotrexate inhibits dihydrofolate reductase leading to nitric oxide synthase uncoupling and enhanced sensitivity of T cells to apoptosis, and hence diminishes immune responses. Three retrospective studies reported favorable effects of methotrexate on relapse rate and disability stabilization in NMOSD patients [96,97,98]. Kitley et al. studied 14 AQP4-IgG-seropositive patients treated with methotrexate at a median dose of 17.5 mg/week plus oral steroid for a median of 22 months, and reported a decrease of median ARR from 1.39 to 0.18 with stabilization or improvement of EDSS in 11 of 14 patients (median EDSS of 5.25 at baseline and 5.0 at last follow-up) [97]. However, with limited experience of its usage in NMOSD and availability of other medications with more evidence of efficacy, methotrexate will not be a commonly use drug for relapse prevention in NMOSD. Cytopenia, including pancytopenia, pneumonitis, and accumulative dose-dependent hepatotoxicity (fatty liver disease, fibrosis, and even cirrhosis) are important complications of methotrexate.

Cyclosporine A, a calcineurin inhibitor, binds to cyclophilins, resulting in calcineurin inhibition and hence inhibition of the translocation of transcription factors (NFAT), leading to reduced transcriptional activation of IL-2, TNFα, IL-3, IL-4, CD40, GM-CSF, and IFNγ, and ultimately to reduced T cell proliferation. It was observed to be effective in the prevention of NMOSD relapse in a retrospective study. Among the 52 AQP4-IgG-seropositive NMOSD patients, nine were treated with cyclosporine A and associated with decrease of ARR from pretreatment, 2.7, to posttreatment, 0.38 (*p* = 0.012) [99]. Tremor, increased hand growth, hypertension, and nephrotoxicity are important side effects of cyclosporine A. Tacrolimus, a macrolide calcineurin-inhibitor with similar mechanisms of action as cyclosporine A, reduces peptidyl-prolyl isomerase activity by binding to immunophilin FKBP-12, and hence leads to the inhibition of T lymphocyte signal transduction and IL-2 transcription. A recent study reported the efficacy of tacrolimus in the relapse prevention of NMOSD. Twenty-five NMOSD patients (88% AQP4-IgG-seropositive) were treated with tacrolimus at 2–3 mg/day (plus oral steroid in 15 patients for >6 months) for a median duration of 11 months; 86% of patients had a decrease in ARR with an improvement in the EDSS score from 4.5 to 2.4 (*p* < 0.0001) [100]. Efficacy of tacrolimus in NMOSD needs to be confirmed by studies of a larger scale in the future. Tremor, hyperglycemia and diabetes mellitus, hyperkalemia, and nephrotoxicity are important side effects of tacrolimus. Both cyclosporine A and tacrolimus increase the risk of infection, including viral, bacterial, and fungal opportunistic infections.

Pulse cyclophosphamide has not been shown to be effective in the prevention of relapse in NMOSD [101].

### 4.2. B Cell Depleting Agents

With the important roles played by B cells in NMOSD pathophysiologies mentioned above, B cell depleting agents have been tried for relapse prevention in NMOSD, initially as off-label use.

#### 4.2.1. Rituximab

Rituximab is a chimeric monoclonal antibody targeting CD20, initially developed for the treatment of B cell lymphoma. It is the first B cell depleting agent used for relapse prevention in NMOSD. It rapidly leads to marked CD20+ B cell depletion via complement-mediated and cell-mediated cytotoxicity. B cell depletion, on average, lasts for 6–9 months, followed by repletion [102]. Quan et al. reported that that rituximab therapy resulted in the depletion of B cells in AQP4-IgG-seropositive NMOSD patients, together with a reversal of the memory B cells to a Breg ratio from a memory B cell predominant state before the therapy towards a Breg predominant state after therapy [42].

Cree et al. first reported eight NMOSD patients refractory to conventional immunosuppressants who had marked disease stabilization (six became relapse free; median ARR declined from 2.6 to 0) and a significant improvement of neurological disabilities (median EDSS score decreased from 7.5 pretreatment to 5.5 at follow-up) with rituximab therapy in an open-label trial [103]. Subsequently, other centers reported an efficacy of rituximab for the prevention of relapses in both AQP4-IgG-seropositive and -seronegative NMOSD patients, either as second- or third-line immunosuppressants after an unsatisfactory response to azathioprine and/or MMF or as first-line immunosuppressant to prevent relapse once NMOSD was diagnosed. Jacob et al. evaluated the use and efficacy of rituximab for treating 25 NMOSD patients (23 adults and 2 children), 23 of whom had relapses despite use of other immunosuppressants. Rituximab was infused at a median interval of 8 months. At a median follow-up of 19 months, the median posttreatment ARR was 0 (range 0–3.2) compared with a pretreatment ARR of 1.7 (0–5.5), *p* < 0.001). Disability improved or stabilized in 20 (80%) patients, and two died during the follow-up period (one from brainstem relapse and one from suspected septicemia). Infections were reported in 20% of patients [104]. Pelkolfer et al. performed a prospective long-term study of 10 patients with NMO treated up to five courses of rituximab as second-line therapy with regular monitoring of B cell counts, and serum concentrations of BAFF, APRIL, AQP4-IgG titer, and immunoglobulin levels. The results showed that rituximab led to sustained clinical stabilization in most patients, and disease activity correlated with B-cell depletion, but not clearly with AQP-IgG titer or the level of APRIL. In addition, the BAFF level increased after the administration of rituximab, and indicated persistent efficacy of rituximab, but did not correlate with disease activity. The investigators concluded that rituximab was well-tolerated even after up to five consecutive treatment courses, with adverse events, including infections such as pneumonia, urosepsis, herpes zoster, and adnexitis, and thrombosis, with one patient who died of cardiovascular failure 3 days after the second infusion of rituximab. The authors recommended that retreatment with rituximab should be applied before the reappearance of circulating B cells [105]. Kim et al. studied the efficacy of rituximab in NMO with 2-year and then 5-year retrospective analyses with induction therapy (rituximab 375 mg/m^2^ weekly for 4 weeks), followed by maintenance therapy (375 mg/m^2^) whenever the frequency of reemerging CD27+ memory B cells in peripheral blood mononuclear cells exceeded 0.05% in the first 2 years and 0.1% thereafter, as measured by flow cytometry [106]. Their 5-year follow-up study of 30 patients showed that 26 patients (87%) exhibited a marked reduction in ARR over 5 years, and a mean pretreatment ARR of 2.4 versus a posttreatment ARR of 0.3. In addition, 18 patients (60%) became relapse free and 28 patients (93%) had disability improved or stabilized after rituximab therapy, without serious adverse events leading to discontinuation. This study supported that repeated rituximab treatment in NMOSD patients over a 5-year period with an individualized dosing schedule according to the frequency of reemerging CD27+ memory B cells led to sustained disease stabilization and clinical benefits without new adverse events [107].

Experience from other centers involving patients of different ethnicities confirmed the above early observations on efficacy of rituximab in NMOSD, as first-line drug or for refractory patients who developed relapse during therapy with other immunosuppressants. Zephir et al. studied the efficacy of rituximab as a first-line drug in NMOSD, and their retrospective analysis of 32 patients (87.5% AQP4-IgG-seropositive) receiving rituximab as a first-line drug showed that after rituximab therapy, 27 patients (84.3%) became relapse free, and the mean ARR decreased from 3.8 to 0.1 (by 97%) and the mean EDSS score decreased significantly from 5.8 to 3.9. In addition, no relevant side effects were observed [108]. French investigators also reported that out a total of 305 NMO patients from a population-based cohort, 21 refractory NMOSD patients were treated with rituximab during a mean follow-up of 31 months; 11 (52.3%) became relapse free with mean ARR decreased from 1.3 to 0.4 (*p* < 0.001) and the median EDSS score improved from 5 to 3 [102].

A recent systematic review (including 46 studies involving 438 patients (381 females) with an age range of 2–77 years, AQP4-IgG-seropositive in 82.7% (320 of 387)) and meta-analysis (including 25 studies that involved 2 or more NMOSD patients treated with rituximab) revealed that rituximab therapy resulted in a mean reduction in ARR ratio of 0.79 and a mean reduction in the EDSS score of 0.64, with a significant correlation between disease duration and EDSS score. Importantly, adverse effects were observed in 114 of 438 (26%) rituximab treated patients, which were minor in most cases, including infusion-related adverse effects (10.3%), infection (9.1%), persistent leukopenia (4.6%), and posterior reversible encephalopathy (0.5%). None of the patients developed PML. Death while receiving rituximab was noted in seven patients (1.6%) [81], but mortality may be due to the natural history of NMOSD, which was reported to be up to 12%. This systematic review and meta-analysis supported that rituximab therapy reduced relapses and neurological disability in NMOSD patients, but the potential adverse effects raised caution for its use as first-line therapy [81]. A similar efficacy of rituximab in the prevention of relapses and a reduction of neurological disability were reported with mild hematological adverse events in five (24%) patients and serious infectious adverse events in four patients (all wheelchair bound at the initiation of rituximab therapy) in a prospective study of 21 Caucasian patients who received at least one cycle of rituximab with follow-up for at least 2 years. The investigators concluded that a fixed treatment regime of rituximab with re-treatment every 6 months was efficacious for NMOSD with a good safety profile. However, to improve the benefit−risk ratio, close monitoring of CD19+ B cells should be performed before re-treatment of patients with severe disability, concomitant leukopenia, and hypogammaglobulinemia [109]. Lebrun et al. analyzed 125 blood samples from 17 rituximab-treated NMOSD patients with measurement of levels of AQP4-IgG and human anti-chimeric antibodies to murine fragment of rituximab, as well as the rituximab concentration. With a mean follow-up time of 7.4 (range 2–16) years and mean interval between rituximab infusions of 9.6 months, all patients improved, with the mean EDSS score decreased from 4 to 2.7. Importantly, the total CD19+ B cell count and residual rituximab concentration did not correlate to re-emergence of CD19+CD27+ memory cells, and the residual rituximab concentration did not correlate with the anti-rituximab antibody production. The investigators concluded that only the CD19+CD27+ memory B cells are a reliable biomarker for biological relapse after rituximab therapy, and its monitoring allowed a decrease in the frequency of rituximab infusion [110].

The first randomized controlled trial of rituximab for NMOSD (RIN-1 study) clarified the efficacy of rituximab for relapse prevention in NMOSD. Tahara et al. performed a multicenter, randomized, double-blind, placebo-controlled clinical trial in Japan. Adult AQP4-IgG-seropostive NMOSD patients aged 16–80 years taking 5–30 mg/day oral steroids with EDSS score of 7.0 or less were recruited for the study, whereas patients on other immunosuppressants were excluded. The studied patients were randomized at 1:1 ratio to rituximab (375 mg/m^2^ weekly for 4 weeks, then two 1000 mg doses separated by 2 weeks 6-monthly at 24 and 48 weeks) or matching placebo, and concomitant oral prednisolone was gradually tapered to 2–5 mg/day. The primary outcome was the time to first relapse, defined as patient-reported symptoms or new signs consistent with CNS lesions and attributable objective changes on MRI or visual evoked potential, within 72 weeks. A total of 38 patients were recruited and randomized to rituximab (*n* = 19) or placebo (*n* = 19). Three in the rituximab group discontinued the study and were analyzed as censored cases. The results showed that seven (37%) of the placebo group relapsed, while none of the rituximab group relapsed (group difference 36.8%, log-rank *p* = 0.0058), and adverse the events identified in more than 20% of patients in either group were infusion reactions, nasopharyngitis, headache, upper respiratory tract infections, and diarrhea (most were mild-to-moderate). Infusion reactions were reported in 37% of patients in the rituximab group, and eight serious adverse events developed (four in three patients (16%) in the rituximab group and four in two patients (11%) in the placebo group). The SAE in the rituximab group included lumbar compression fracture and infection around the nail of the right foot, diplopia, and uterine cancer. All patients with SAE recovered and no deaths were reported. The investigators interpreted that rituximab prevented relapses for 72 weeks in AQP4-IgG-seropositive NMOSD patients, and despite the limitations of the small sample size and mild disease activity of the studied patients, the results suggested that rituximab could be a useful maintenance therapy for AQP4-IgG-seropositive patients [80].

Rituximab has been used to prevent relapse in NMOSD on an off-label basis for more than 15 years as both a first-line agent or second/third agents in patients with an unsatisfactory response to other immunosuppressants, typically azathioprine and/or MMF. Rituximab has been recommended as a first-line maintenance treatment of NMOSD by in the 2010 guidelines from the European Federation of Neurological Societies [111] and the 2014 recommendations of the Neuromyelitis Optica Study Group [69]. There is no standardized rituximab treatment protocol in NMOSD, and in clinical practice, its use is driven by the experience of the individual neurologist and center. There is a general consensus that the induction phase should be infusion of 2 g during two 1 g doses separated by 2 weeks or 375 mg/m^2^ weekly for 4 weeks. The maintenance regimen is controversial—different protocols are used in different centers, including the reinfusion of rituximab (375 mg/m^2^) when CD27+ memory B cell frequency is at least 0.05% in PBMCs (85,86), or two 1 g doses separated by 2 weeks every 6–9 months, or when CD19+ B cell frequency wisas greater than 0.1% [105], or every 6–9 months based on clinical status and patients’ preference [112], or a 100 mg infusion once a week for 3 weeks depending on circulating B cell repopulation [113]. In this last study involving 30 Chinese MS or NMOSD patients treated with rituximab, the mean duration after 100 mg dose of rituximab until the CD19+ B cell frequency was greater than 2% was 99 days (range 43–172) compared with 184 days after a 1000 mg dose of rituximab [113]. Individual NMOSD patients with an early and late repopulation of B cells after rituximab therapy were observed [81]. In the absence of a consensus, it has been recommended to (1) perform monitoring of CD19+ B cells every 3 months and reinfuse rituximab when CD19+ B cells become detectable, as this is feasible in most centers, contrary to the monitoring of CD19+CD27+ memory B cells, which requires the detection of very few cells and needs technique standardization of flow cytometry, and to (2) administer 1 g as the dose for maintenance therapy, as it seems to be a good compromise for preventing underdosing therapy [114]. Repeated infusions every 6 months or upon B cell repletion are associated with an optimal outcome [69,115]. Overall, 60–80% of patients will avoid relapse as long as B cell depletion is maintained, and those having no detectable circulating B cells at the time of relapse should be switched to alternative therapies [10]. New biomarkers such as the FcR3A-158F allele may impact rituximab therapeutic strategy in the future [116].

Overall, rituximab is well tolerated in autoimmune diseases, including NMOSD. The main side effects are infusion reactions and infections (opportunistic and nonopportunisitic). Infusion reactions are common and can usually be managed by pretreatment with intravenous steroid, antihistamine, and slow infusion. Various infections have been reported, mostly herpes zoster and tuberculosis, and rarely PML. The risk of PML in rheumatoid arthritis treated with rituximab is calculated to be 1/25,000 [117]. Hepatitis B, active tuberculosis, and other severe infections need to be excluded or treated before the initiation of treatment [10]. Hypogammaglobulinaemia can develop especially with prolonged therapy in about 20%, which can lead to an immunosuppressed state if severe, and also, rarely, early or delayed neutropenia [118].

Mealy et al. performed a retrospective multicenter study to compare the efficacy of azathioprine, MMF, and/or rituximab in 90 patients with NMO/NMOSD, in which treatment failure was defined as any new inflammatory CNS events that occurred despite immunosuppressive treatment. The results showed that azathioprine reduced ARR by 72.1% with a 53% failure rate, MMF reduced ARR by 87.4% with a 36% failure rate, and rituximab reduced ARR by 88.2%, with two-thirds of patients achieving complete remission. This study suggested that initial treatment with rituximab; MMF; and, to a lesser extent, azathioprine, significantly reduced the relapse rate in NMOSD; and those who failed initial treatment often achieved remission when switched from one to another of these drugs [88]. Another retrospective study of 138 NMOSD patients treated with azathioprine, MMF, and rituximab using primary outcome measures of ARR, annualized severe relapse rate (ASRR), and the time to first relapse and time to first severe relapse yielded consistent findings. A comparison among the groups revealed that azathioprine had a significantly higher risk of relapse relative to rituximab (HR: 1.82, *p* = 0.03), and the HR for severe relapse for azathioprine and MMF compared with rituximab were 11.66 (*p* = 0.001) and 5.96 (*p* = 0.048), respectively. In addition, the time to first relapse and first severe relapse were also significantly different between the groups, and five patients who failed to respond to MMF became relapse free after switching to rituximab. This study provided evidence that rituximab is more effective than azathioprine in relapse prevention, and more effective than MMF in severe relapse prevention in NMOSD [119].

#### 4.2.2. Inebulizumab

Inebulizumab is a humanized monoclonal antibody targeting CD19, and depletes CD19+ B cells, as well as CD19-expressing plasmablasts and plasma cells, which produce and secrete AQP4-IgG; it has B cell suppressive and anti-inflammatory effects, as well as an inhibitory effect on AQP4-IgG synthesis and secretion from antibody secreting cells [84]. Inebulizumab was shown to be effective at preventing relapse in a double-blind randomized placebo-controlled clinical trial [84]. NMOSD patients, AQP4-IgG seropositive and seronegative, with an EDSS score of 8.0 or less, and at least one relapse requiring rescue therapy in the previous 1 year or at least two relapses in the previous 2 years were recruited. Patients were randomized at a 3:1 ratio to either inebulizumab (300 mg) or placebo. Inebulizumab or placebo were administered on days 1 and 15. As B cell depletion may be associated with acute attack upon treatment initiation, all participants received oral prednisolone 20 mg daily, or equivalent corticosteroid between days 1 and 14, tapered to day 21, to minimize the risk of relapse immediately following the first inebulizumab treatment. Other immunosuppressants were not allowed during the randomized controlled period, which was up to 197 days or until the occurrence of an adjudicated attack. At the end of the randomized controlled period, all participants were offered participation in the open-label period to maintain/establish B-cell depletion at a dose of inebulizumab of 300 mg every 26 weeks, with a continuation of safety follow-up from 12 months after the last dose [84].

The primary endpoint was time to an NMOSD attack, determined by an adjudicated committee. In total, 230 patients were recruited, with 174 randomized to inebulizumab while 56 were assigned to placebo. The randomized controlled period was terminated before the completion of enrolment due to a clear demonstration of efficacy. Twenty-one (12%) of the 174 patients of the inebulizumab arm had an NMOSD attack versus 22 (39%) of the 56 patients of the placebo arm (hazard ratio 0.272; *p* < 0.0001), and the number needed to treat was 3.73. Among the AQP4-IgG-seropositive patients, 18 (11%) of the 161 participants receiving inebulizumab had an attack versus 22 (42%) of the 52 receiving the placebo (HR 0.227; *p* < 0.0001), and the number needed to treat was 3.23. Among the 17 AQP4-IgG-seronegative participants (13 randomized to inebulizumab and 4 to placebo), three had an attack and all three received inebulizumab. As only four participants were randomized to the placebo, efficacy could not be interpreted in the AQP4-IgG-seronegative group. Analysis of the secondary endpoints revealed a lower frequency of EDSS score worsening from baseline, reduced likelihood to experience optic neuritis, and lower numbers of cumulative active MRI lesions and NMOSD-related hospitalizations with inebulizumab therapy compared with the placebo. This study also confirmed that the efficacy of inebulizumab was consistent across the clinical presentation of myelitis and optic neuritis domains, when only attacks positively adjudicated by unanimous decision of the independent adjudication committee were considered; when all investigator-suspected attacks were considered; and, importantly, in different ethnic groups (white, non-white, Asian, and non-Asian). The immunological effects of inebulizumab were observed within 4 weeks, with a significant depletion of circulating B cells compared with the placebo (B cell counts dropped to less than 10% of baseline), which persisted during the randomized controlled period.

Serious adverse events were similar among both the inebulizumab and placebo groups (5% and 9%, respectively). Two deaths occurred in the open-label period. One patient randomized to placebo succumbed after a severe NMOSD attack preceded by pneumonia, which developed shortly after randomization. He received the first day-1 open-label period dose of 300 mg inebulizumab, but died at home 9 days later. His death was not considered to be treatment-related. The other patient was randomized to inebulizumab and received the day-1 and day-15 doses, and then entered the open-label period after an adjudicated attack. The participant developed new-onset weakness, aphasia, neurological decline, and seizures, with an MRI showing large new lesions over white-matter and gray-matter structures that were not suggestive of PML. CSF was negative for JCV by PCR and brain biopsy was not performed. The participant succumbed after respiratory arrest. A definitive diagnosis was not possible and differential diagnoses include ADEM, atypical NMOSD attacks, and PML. The possibility of treatment-related death could not be excluded for this participant.

This study provides evidence that inebulizumab reduced the risk of NMOSD attacks compared with placebo; hence, inebulizumab has potential as an evidence-based treatment for AQP4-IgG-seropositive NMOSD patients.

Inebilizumab was approved for relapse prevention in adult patients with AQP4-IgG-seropositive NMOSD by the FDA in 2020 [120]. As B-cell depleting therapies are associated with an increased risk of malignancy and infection, including PML, the long-term safety of inebulizumab in the treatment of NMOSD requires long-term observation and analysis of large-scale studies.

### 4.3. Blockade of Interleukin-6 Signaling

IL-6 is an important proinflammatory cytokine in NMOSD pathophysiologies, as mentioned above. Chihara et al. reported that peripheral blood plasmablasts increased in AQP4-IgG-seropositive NMOSD patients, and IL-6 drove these plasmablasts to produce and secrete AQP4-IgG, whereas blocking the IL-6 receptor signaling by IL-6 receptor antibody reduced the survival of plasmablasts in vitro [34]. Serum and CSF IL-6 levels were increased in the NMOSD patients compared with the healthy subjects, especially during relapse [30].

#### 4.3.1. Tocilizumab

About 20% of NMOSD patients develop relapse, despite complete depletion of the B cells by rituximab [104,105,106]; the agents that block the IL-6 pathway and hence its signaling activity have been considered in NMOSD. Ayzenberg et al. reported their experience with tocilizumab, a humanized antibody targeting the IL-6 receptor, in three female patients (median age 39, range 26–40 years) with aggressive AQP4-IgG-seropositive NMOSD uncontrolled by other immunsuppressants and despite complete CD20-cell-depletion during rituximab therapy (median ARR 3.0 and EDSS score increased from 5.0 to 6.5). After switching to tocilizumab, the median ARR decreased from 3.0 (range 2.3–3.0) to 0.6 (range 0–1.3), but there was no change in clinical disability. This study suggested that tocilizumab could be effective in therapy-resistant NMO patients [121]. Consistent efficacy of tocilizumab in NMOSD patients nonresponsive to rituximab were also reported by other investigators [122,123]. Ringelstein et al. evaluated the long-term safety and efficacy of tocilizumab in NMO and NMOSD. In this retrospective study, eight Caucasian highly active AQP4-IgG-seropositive patients with NMO (*n* = 6) or NMOSD (*n* = 2) with disease refractory to previous immunosuppressants, including rituximab, were switched to tocilizumab (6–8 mg/kg of body weight per dose monthly). The mean follow-up duration after switching tocilizumab was 30.9 months. The results showed that during tocilizumab therapy, the median ARR decreased significantly from 4.0 to 0.4, and the median EDSS score significantly decreased from 7.3 to 5.5, paralleled by the decrease in the number of patients with active MRI lesions from 6 to 1. Three patients remained relapse free during tocilizumab therapy. In addition, the AQP4-IgG titers and pain levels decreased significantly during tocilizumab therapy. Adverse effects included cholesterol rise in six patients, infection in for, and deep venous thrombosis and neutropenia in one each [123]. The investigators concluded that prolonged tocilizumab therapy might be safe and effective from early treatment phases onward for refractory highly-active NMOSD.

Zhang et al. performed an open-label, multicenter, randomized phase 2 trial at six hospital in China to study the safety and efficacy of tocilizumab versus azathioprine in highly relapsing NMOSD (TANGO study). Adult patients aged 18 years or older with highly relapsing NMOSD (at least two clinical relapses in previous 12 month, or three relapses in the previous 24 months with at least one relapse within the previous 12 months) with an EDSS score of 7.5 or less were randomized (1:1) to intravenous tocilizumab (8 mg/kg every 4 weeks) or oral azathioprine (2–3 mg/kg daily). The investigators and patients were aware of the treatment allocation, while the central review committee, EDSS raters, and radiologists were masked to the allocation. The minimum planned treatment duration was 60 weeks after randomization. The primary outcome was time to first relapse. A total 118 patients were recruited, with 59 randomized to the tocilizumab arm and 59 to the azathioprine arm. The results showed that the median time to first relapse was longer in the tocilizumab group than the azathioprine group (78.9 weeks versus 56.7 weeks, *p* = 0.0026), and 14% of the tocilizumab group had a relapse at the end of the study compared with 47% of the azathioprine group (HR 0.236, *p* < 0.0001). In the per-protocol analysis, 89% of the tocilizumab group were relapse-free compared with 56% of the azathioprine group (HR 0.188, *p* < 0.0001), and the median time to first relapse was longer in the tocilizumab group compared with the azathioprine group (67.2 weeks versus 38.0 weeks, *p* < 0.0001). Treatment-associated adverse events were reported in 61% and 83% in the tocilizumab and azathioprine groups, respectively. There was no treatment-related death in both groups. The investigators interpreted that tocilizumab significantly reduced the risk of relapse compared with azathioprine in NMOSD. Tocilizumab is a potentially effective and safe treatment for relapse prevention in NMOSD [82].

#### 4.3.2. Satralizumab

Satralizumab is a subcutaneously administered humanized monoclonal antibody targeting the interleukin-6 (IL-6) receptor. It binds to both the membrane-bound and soluble IL-6 receptors and prevents the binding of IL-6, hence blocking IL-6 signaling pathways that are involved in inflammation [124]. Satralizumab is designed to dissociate from the antigen in a pH-dependent manner and be released to the peripheral blood for repeated antigen binding, hence prolonging its elimination half-life in plasma. Yamamura et al. performed a phase 3, randomized, double-blind, placebo-controlled trial to study the efficacy of satralizumab added to immunosuppressant treatment in NMOSD patients (SAkuraSky). AQP4-IgG-seropositive and -seronegative NMOSD patients aged 12 to 74 years with active disease (at least two relapses in the previous 2 years before screening, with at least one relapse in the previous 12 months) and an EDSS score of 0 to 6.5 were randomized in a 1:1 ratio to receive either satralizumab (120 mg) or placebo subcutaneously at weeks 0, 2, and 4, and every 4 weeks thereafter, added to stable immunosuppressant treatment. Exclusion criteria included previous treatment with the agent targeting IL-6 pathway, alemtuzumab, total body irradiation or bone marrow transplantation, use of eculizumab or belimumab, or multiple sclerosis DMT 6 months before baseline, and the use of anti-CD4 agents, cladribine, or mitoxantrone within 2 years before baseline. The primary endpoint was the first protocol-defined relapse in a time-to-event analysis. The major secondary endpoints were the change from baseline to week 24 in the visual-analogue scale pain score and the Functional Assessment of Chronic Illness Therapy-Fatigue (FACIT-F) score.

A total of 83 patients were recruited, with 41 randomized to the satralizumab arm and 42 to the placebo arm. The median treatment durations with satralizumab and placebo in the double-blind period were 107.4 weeks and 32.5 weeks, respectively. Protocol-defined relapse occurred in eight patients (20%) receiving satralizumab versus 18 (43%) receiving placebo (HR, 0.38; 95% CI, 0.16–0.88, adjusted *p* = 0.02). The percentages of patients free from relapse at 48 weeks was 89% and 66% in the satralizumab and placebo groups, respectively, and these values at 96 weeks were 78% and 59%, respectively. Among the 55 AQP4-IgG-seropositive patients, relapse occurred in 3 of 27 (11%) patients receiving satralizumab versus in 12 of 28 (43%) receiving placebo (HR, 0.21; 95% CI, 0.06–0.75) while among 28 AQP4-IgG-seronegative patients, relapse occurred in 5 of 14 (36%) and 6 of 14 (43%) in the satralizumab and placebo groups, respectively (HR 0.66; 95% CI, 0.20 to 2.24). The subgroup analysis suggested that satralizumab led to a lower risk of relapse than placebo in AQP4-IgG-seropositive patients, but there was insufficient evidence to indicate a lower risk of relapse in the AQP4-IgG-seronegative patients. There were no significance differences in either the VAS pain score or the FACIT-F score. Importantly, rates of serious adverse events and infections were indifferent between the satralizumab and placebo groups [85]. The investigators concluded that satralizumab added to immunosuppressant therapy in NMOSD patients led to a lower risk of relapse than the placebo, particularly among AQP4-IgG-seropositive patients, but showed no benefit on pain and fatigue compared with the placebo. Further trials of a large-scale and longer duration are recommended to determine the efficacy, durability, and safety of satralizumab in NMOSD, and to investigate its effect compared with other treatments.

Satralizumab was also studied in a phase-3 study evaluating its efficacy and safety as a monotherapy (SAkuraStar study), which showed a similar efficacy with a significant reduction in the time to the first relapse and relapse risk in AQP4-IgG-seropositive NMOSD patients with active disease [86]. The frequency of serious adverse events was similar in the treatment and control arm, without anaphylactic reactions, opportunistic infections, or deaths in both studies. The FDA approved satralizumab for the treatment of adult patients with AQP4-IgG-seropositive NMOSD including by self-injection in 2020.

### 4.4. Blocking of Complement Activation and Complement-Mediated Pathologies

#### Eculizumab

Eculizumab is a monoclonal antibody that inhibits the terminal complement component C5, and prevents its cleavage into C5a and C5b. C5a is proinflammatory and mediates the chemotaxis of leukocytes to the inflammatory sites, whereas C5b, the terminal complement component, coordinates the formation of the membrane attack complex (MAC), which is cytotoxic to astrocytes in NMOSD [125]. The efficacy of eculizumab in the prevention of relapse in NMOSD was initially suggested by a small scale open-label clinical trial of 14 AQP4-IgG-seropositive NMOSD patients with highly active disease [126]. Pittock et al. performed a randomized controlled trial that confirmed the efficacy of eculizumab for the reduction of risk of relapse in AQP4-IgG-seropositive NMOSD [83]. In this randomized, double-blind, time-to-event trial, adult patients (18 years or older) with active AQP4-IgG-seropositive NMOSD (at least two relapses in the previous 12 months, or two relapses in the previous 24 months with at least one relapse that occurred within the previous 12 months) and an EDSS score of 7 or less were randomized in a 2:1 ratio to intravenous eculizumab (900 mg weekly for 4 weeks followed by 1200 mg every 2 weeks) or matched placebo. All of the participants were vaccinated against Neisseria meningitides before receiving a trial agent, and the continued use of stable-dose of immunosuppressants was permitted. Patients who had received rituximab or mitoxantrone in the previous 3 months, IVIg in the previous 3 weeks, prednisone at doses greater than 20 mg daily or equivalent for other corticosteroids at screening, and those with unresolved meningococcal disease or systemic bacterial or other significant infections not treated with appropriate antibiotics were excluded from the study. The primary endpoint was the first adjudicated relapse, and secondary endpoints included the adjudicated annualized relapse rate, changes in EDSS score, modified Rankin scale, and Hauser Ambulation Index.

The trial was terminated after 23 of the 24 planned adjudicated relapses with the uncertainty in estimating when the final event would occur. Ninety-six patients were randomized to receive eculizumab and fourty-seven to the placebo. The mean ARR in the previous 24 months was 1.99, and 76% of the patients continued their previous immunosuppressants during the trial, while 24% of patients did not take any concomitant immunosuppressants during the trial. The results showed that adjudicated relapses occurred in 3 of 96 patients (3%) and 20 of 47 (43%) in the eculizumab and placebo arms, respectively (HR 0.06; *p* < 0.001); the median time to the first adjudicated relapse was not reached in the eculizumab arm and reached at 103 weeks in the placebo arm. The adjudicated ARR was 0.02 in the eculizumab arm versus 0.35 in the placebo arm (rate ratio 0.04; *p* < 0.001), with the majority of relapses being myelitis. Subgroup analysis revealed that the efficacy of eculizumab at reducing the risk of relapse was consistent, irrespective of any concomitant immunosuppressants and whether adjudicated relapses or physician-determined relapses were used for the analysis. However, there was no significant difference in disability progression, as shown by the change in EDSS score and the mean change of −0.18 in the eculizumab arm versus 0.12 for the placebo arm (least-squares mean difference, −0.29; 95% CI −0.59 to 0.01). Upper respiratory tract infections and headaches were more common in the eculizumab arm, with one death from pulmonary empyema. One patient in the eculizumab arm who was receiving concomitant azathioprine died from pulmonary empyema (cultures yielded *Streptococcus intermedius* and Peptostreptococcus micros) after 108 weeks in the trial. No patients developed meningococcal infection during the trial. This trial concluded that eculizumab was associated with a lower risk of relapse compared with the placebo in AQP4-IgG-seropositive NMOSD, but no benefit on disability progression; further study is required to clarify the long-term effects of eculizumab in NMOSD patients [83].

Eculizumab was approved by the FDA as treatment to prevent relapse in AQP4-IgG-seropositive adults with NMOSD in 2019, followed by the European Union and in Japan. Adverse effects include infection, especially by encapsulated bacteria and upper respiratory tract infections. Immunization with the meningococcal vaccine is mandatory before the initiation of therapy, at least 2 weeks before the first dose of eculizumab. In the USA, a compulsory program to evaluate and mitigate the risk of infection associated with eculizumab therapy has been instituted [10].

## 5. Long-Term Intermittent Intravenous Immunoglobulins for Prevention of Relapse

Intravenous immunoglobulins (IVIg) have been reported to be effective to reduce the relapse rate in NMOSD and for the improvement of neurological disability in case series. Magraner et al. reported on eight NMOSD patients (25% AQP4-IgG-seropositive) treated with IVIg (0.7 g/kg/day for 3 days, 4–21 infusions per patient) for an mean treatment duration of 19 months. The mean ARR decreased significantly from 1.8 to 0.0006 and the EDSS score reduced significantly from 3.3 to 2.6 [127]. Viswanathan et al. studied the response of six NMOSD patients (four AQP4-IgG-seropositive and two unknown antibody status) to IVIg (0.4 g/kg/day for 5 days, then 0.4–1.0 g/kg/day every 2 to 3 months) treatment for a median duration of 4 years. The median ARR decreased significantly from 0.75 to 0.15, while the median EDSS score remained stable at 6.5, and half of the patients were relapse-free at the last follow-up [128]. IVIg is probably a safe treatment for relapse prevention in NMOSD, but efficacy needs to be confirmed in controlled trials. It can be considered in NMOSD patients with repeated infections with immunosuppressant therapy.

## 6. Treatment for AQP4-IgG-Seronegative NMOSD Patients

Diagnosis of patients who fulfil the 2015 diagnostic criteria for AQP4-IgG seronegative NMOSD patients requires caution. The AQP4-IgG seronegative status needs to be confirmed with cell-based assays, ideally repeated at least two to three times in durations of 6–9 months [2,129], and those who are AQP4-IgG-seronegative should be assayed for MOG-IgG by cell-based assays (ideally live-cells) to look for myelin-oligodendrocyte glycoprotein antibody associated disease (MOGAD). Patients who are seronegative for both AQP4-IgG and MOG-IgG (double-seronegative NMOSD) pose treatment difficulty, as the recent randomized placebo-controlled clinical trials of eculizumab, inebulizumab, and satralizumab either did not include such patients (eculizumab) or failed to provide evidence that the newer agents are effective for relapse prevention (inebulizumab and satralizumab) in these double-seronegative patients. The Spanish NMO Study Group reported that double-seronegative and AQP4-IgG-seropositive NMOSD patients had a similar clinical outcome, while those seropositive for MOG-IgG had a better prognosis [130]. In a study of 67 patients, French investigators observed that MMF as a first-line therapy was effective in relapse prevention and disability stabilization/improvement in NMOSD patients (based on 2015 diagnostic criteria), irrespective whether they were AQP4-IgG seropositive, MOG-IgG seropositive, or double-seronegative [79]. A recent multicenter retrospective study of 245 NMOSD patients reported that rituximab and MMF were equally effective in AQP4-IgG-seropositive and double-seronegative NMOSD patients [78].

## 7. Treatment Issues for Pregnancy

The mouse and human placenta express AQP4 and are susceptible to potential AQP4-IgG mediated placentitis, which, together with fetal death, have been demonstrated in mice injected with human AQP4-IgG [131]. NMOSD patients, especially AQP4-IgG-seropositive, have a greater risk of spontaneous miscarriage than healthy individuals [132], and the relapse rate is higher in the postpartum period and possibly during pregnancy [133,134]. An association between NMOSD disease activity and poor pregnancy outcome have been shown by retrospective studies and case reports [135,136]. AQP4-IgG-seropositive NMOSD patients have a high risk of complications, especially with active disease during or just before pregnancy, and not on prophylactic immunosuppressive therapy. Several reports described good outcomes of neonates and NMOSD mothers who received adequate immunosuppressive treatment or plasma exchange. IgG crosses the placenta barriers during the second and third trimester, AQP4-IgG is likely to be transferred to the fetus, and high AQP4-IgG levels have been detected in the umbilical cord blood in infants of mothers with active NMOSD during pregnancy, but became seronegative by 1–3 months of age and none of the infants had clinical signs of NMOSD [137].

The immunosuppressive medications for the prevention NMOSD relapse mentioned above have different safety and risk concerns for use during pregnancy. The details are beyond the scope of the review, and reference to an excellent review is recommended (Mao-Draayer et al., 2020). The following recommendations are useful in counselling of women with NMOSD seeking pregnancy [137].

I. Before pregnancy, patients should understand that pregnancy is risky with NMOSD, and early interdisciplinary discussion between neurologist and gynecologist is recommended. MMF, methotrexate, and mitoxantrone should be stopped before conception, and switching to safer treatments such as azathioprine or rituximab/ocrelizumab should be considered. Planning to conceive close to the last rituximab dose is recommended. For patients on rituximab and ocrelizumab treatment, re-dose if pregnancy is unsuccessful after 6 months.
II. During pregnancy, continuation of azathioprine, eculizumab, or tocilizumab during pregnancy can be considered depending on disease activity before pregnancy. Check B cell counts in newborn of patients on B cell depleting agents during pregnancy, this helps to plan vaccination of newborn. Delivery should be in hospitals specializing in high-risk pregnancies with special pediatric care. If a patient develops a relapse, non-fluorinated glucocorticoid treatment, plasma exchange, or immunoadsorption can be considered.
III. In the postpartum period, newborns should be evaluated for possible symptoms due to transferred antibodies or medications. Breast-feeding should be decided after a careful personalized risk−benefit evaluation. If no breastfeeding is planned, restart NMOSD treatments that have been stopped before pregnancy shortly after delivery, and for mothers taking monoclonal antibodies, breastfeeding in preterm babies is not recommended until more data become available. For those who breastfeed and with severe NMOSD, consider continuation or restart of azathioprine or monoclonal antibodies with careful monitoring. Glucocorticoid (wait 1–4 h after dose), plasma exchange, or immunoadsorption are treatment options if relapse occurs.

## 8. Potentially Effective Treatments and New Directions

### 8.1. Plasma Cell Depleting Agent

AQP4-IgG are produced by ASC, including plasma cells, which can be long-lived in the bone marrow to produce autoantibodies for years. A recent study evaluated the safety and efficacy of bortezomib, a selective inhibitor of the 26S proteasome subunit in patients with highly relapsing NMOSD. This longitudinal study recruited five Chinese female NMOSD patients who had at least two relapses in previous 6 months or three relapses throughout the years despite treatment with various immunosuppressants including prednisolone, azathioprine, rituximab, or cyclophosphamide. The participants received four cycles of subcutaneous bortezomib at a dosage of 1 mg/m^2^ of the body surface area on days 1, 4, 8, and 11 per cycle, followed by a 10-day treatment-free interval with concomitant oral steroid or azathioprine. After initiating bortezomib, four of the five patients became relapse-free during the 1-year follow-up. No patient experienced further neurological deterioration at the end of the study, and the median EDSS scores reduced from 5.5 at baseline to 3.5 after 1-year follow-up associated with an improvement in the pain scale. Adverse effects were mild and transient. In addition, serum AQP4-IgG titer, peripheral blood CD19+ B cells, and CD138+ plasma cells decreased significantly after bortezomib treatment. This study suggested that bortezomib could be a promising escalating therapy for highly active NMOSD refractory to or intolerant of current immunosuppressants by depleting long-lived plasma cells [138].

### 8.2. Autologous Nonmyeloablative Hematopoietic Stem Cell Transplantation

Burt et al. studied 13 NMOSD patients (11 AQP4-IgG-seropositive and 1 with neuropsychiatric SLE) treated with autologous nonmyeloablative stem cell transplantation using a regime consisting of cyclophosphamide and rATG (thymoglobulin) and rituximab with the infusion of unselected peripheral blood stem cells. After a median follow-up duration of 57 months, the patient with coexisting SLE died of complications of active SLE; for the other 12 NMOSD patients, 11 were more than 5 years post-transplant and 80% were relapse-free off all immunosuppressants. In addition, at 1 and 5 years after transplant, the EDSS score improved from a baseline mean of 4.4 to 3.3 (*p* < 0.01) at 5 years, together with improvements in the Neurological Rating Scale and the quality of life (Short-Form-36) scores. Interestingly and importantly, nine patients converted to AQP4-IgG-seronegative and complement-activating and cell-killing ability was lost in six of seven patients with pre- and post-transplant testing. Two remained AQP4-IgG-seropositive and relapsed within 2 years of transplant, while no patient with seronegative conversion relapsed. The investigators concluded that prolonged drug-free remission with conversion to AQP4-IgG seronegativity following nonmyeloablative hematopoietic stem cell transplantation warrants further study [139].

### 8.3. Bruton’s Tyrosine Kinase Inhibitor

Brutons’s tyrosine kinase (BTK) is an enzyme involved in B cell activation upon antigen recognition by B cell receptor (BCR), which is the primary sensor to initiate signaling. In addition, BTK also has a crucial role in Fc receptor (FcR) mediated activation of myeloid cells, which facilitate antigen recognition via antibody-mediated opsonization [140]. BTK inhibitors (BTKi) inhibit B cell activation without depletion of B cells, typically by CD20 monoclonal antibodies (rituximab and ocrelizumab). Prolonged and repeated B cell depletion may lead to opportunistic infections, including life threatening PML [114,117]. BTKi are being developed as therapeutic agents for multiple sclerosis. Convincing results are being obtained with phase 2 clinical trials [141] and phase 3 trials are on-going. BTK inhibitor may be a novel effective agent for relapse prevention in NMOSD.

### 8.4. Future Treatment Alternatives in Preclinical Phase

Remyelination or myelin repair: this can be beneficial in an inflammatory demyelinating disorder such as NMOSD. A logical approach in this direction is to promote differentiation and proliferaton of oligodendrocyte precursor cells (OPC) to mature oligodendrocytes capable of myelination. Clobetasol, an approved drug, was shown to promote OPC differentiation in cultured cells and to induce remyelination in mouse brain with AQP-IgG and complement-induced injury [142].

Induction of immune tolerance: a potential cure for an autoimmune disease may be achieved by the induction of immune tolerance to the autoantigen by vaccination. This definitely deserves continued research, as the majority of NMOSD patients have underlying AQP4 autoimmunity with the autoantigen clearly defined [143].

## Figures and Tables

**Table 1:** Treatments for acute attacks of neuromyelitis optica spectrum disorders.

Intravenous methylprednisolone Plasmapheresis Intravenous immunoglobulins Immunoadsorption

**Table 2:** Treatments for relapse prevention in neuromyelitis optica spectrum disorders.

Conventional Immunosuppressants Corticosteroids Azathioprine Mycophenolate mofetil Methotrexate Cyclosporine A Tacrolimus Mitoxantrone
B cell depleting agents Rituximab Inebulizumab
Interleukin-6 signaling blocking agents Tocilizumab Satralizumab
Complement blocking agents Eculizumab
Intravenous immunoglobulins

**Table 3:** Core studies of treatments for neuromyelitis optica spectrum disorders.

Treatment	Study Design	Main Outcomes
Oral prednisolone [75]	R	lower ARR
Azathioprine [76,77]	R	lower ARR, EDSS stable or improved in 61–78%
Mycophenolate mofetil [78,79]	R	lower ARR, EDSS stable or improved in 83–91%
Rituximab		
RIN-1 study [80]	RCT	no relapse in rituximab arm versus 7 relapses in control arm at 72 weeks (group difference 36.8%, *p* = 0.0058)
[81]	M	mean 0.79 reduction in mean ARR ratio, mean 0.64 reduction in mean EDSS score at mean follow-up duration of 27.5 months
Tocilizumab [82]	P	tocilizumab prolongs time to first relapse versus azathioprine (78.9 weeks versus 56.7 weeks), lower risk of relapse in tocilizumab arm at 60 weeks
Eculizumab [83]	RCT	eculizumab reduces risk of relapse by 94.2% at week 48; 96.4% free of relapse in eculizumab arm versus 51.9% in control arm at 96 weeks; no difference in EDSS progression in the two arms
Inebulizumab [84]	RCT	inebulizumab reduces risk of relapse by 77% in AQP4-IgG-seropositive patients at 28 weeks, significant benefits in EDSS prgression, NMOSD-related hospitalizations and number of active MRI lesions
Satralizumab		
add-on therapy [85]	RCT	satralizumab reduces risk of relapse by 62%; 78% relapse free (satralizumab) versus 59% (control) at 96 weeks; relapse risk reduction of 79% in AQP4-IgG-seropositive patients and 34% in seronegative patients
monotherapy [86]	RCT	satralizumab reduces relapse risk by 55%; 72% and 51% relapse free at 96 weeks in satralizumab and control arms respectively; relapse risk reduction 74% in AQP4-IgG-seropositive patients versus 19% in seronegative patients

M = metanalysis; P = non-randomized prospective study; R = retrospective case series/studies; RCT = randomized controlled trial; Reference number in bracket.
